# Music Performance Anxiety: Can Expressive Writing Intervention Help?

**DOI:** 10.3389/fpsyg.2020.01334

**Published:** 2020-06-16

**Authors:** Yiqing Tang, Lee Ryan

**Affiliations:** ^1^Fred Fox School of Music, The University of Arizona, Tucson, AZ, United States; ^2^Department of Psychology, The University of Arizona, Tucson, AZ, United States

**Keywords:** music performance anxiety, piano playing, expressive writing intervention, performance quality, self-talk

## Abstract

Performance is an essential part of music education; however, many music professionals and students suffer from music performance anxiety (MPA). The purpose of this study was to investigate whether a 10-min expressive writing intervention (EWI) can effectively reduce performance anxiety and improve overall performance outcomes in college-level piano students. Two groups of music students (16 piano major students and 19 group/secondary piano students) participated in the study. Piano major students performed a solo work from memory, while group/secondary piano students took a sight-reading exam of an eight-measure piano musical selection. All students performed twice, at baseline and post-EWI, with 2 or 3 days between performances. During the EWI phase, students were randomly divided into two groups: an expressive writing group and a control group. Students in the expressive writing group wrote down feelings and thoughts about their upcoming performances, while students in the control group wrote about a topic unrelated to performing. Each student’s pulse was recorded immediately before performing, and each performance was videotaped. Three independent judges evaluated the recordings using a modified version of the Observational Scale for Piano Practicing (OSPP) by Gruson (1988). The results revealed that, by simply writing out their thoughts and feelings right before performing, students who had high MPA improved their performance quality significantly and reduced their MPA significantly. Our findings suggest that EWI may be a viable tool to alleviate music performance anxiety.

## Introduction

Do classical music lovers ever notice that their presence at concert venues tends to raise heart rates and trigger anxiety in performing musicians? Performance anxiety is a complex phenomenon and has been studied in a variety of contexts, including sports competitions (Patel et al., 2010), public speaking (Beatty and Behnke, 1991), test-taking (Ramirez and Beilock, 2011), job interviews (McCarthy and Goffin, 2004), and performing arts (Williamon, 2004). For musicians, being able to perform high-quality concerts consistently under pressure is critical to their career’s success and length (Kenny, 2011; Williamon et al., 2013). Coping with performance anxiety is a constant battle even for the most accomplished musicians and performers (McGinnis and Milling, 2005). For student-musicians, anxiety about performing their best can be extremely high, particularly in performance-evaluative situations.

Music performance anxiety (MPA) has been defined as “the experience of marked and persistent anxious apprehension related to musical performance” (Kenny, 2010, p. 433). Several interactive factors comprise this form of anxiety, including genetics; environmental stimuli; and an individual’s experience, emotions, cognition, and behaviors, may cause musicians to experience MPA to varying degrees (Kenny, 2011). Indeed, for musicians, performing in public is a demanding activity and can cause considerable stress (Williamon et al., 2013). Even for highly skilled and experienced performers, performing a concert in public is stressful (Eysenck et al., 2007; Nieuwenhuys and Oudejans, 2012). Research showed that an expert concert-pianist who performed regularly around the world demonstrated an intensive autonomic arousal by loss of complexity in cardiovascular response temporarily when performing in front of a large crowd (Williamon et al., 2013). Although MPA symptoms vary widely, including elevated heart rate, sweaty palms, disrupted concentration, diminished confidence, and negative thoughts (Wan and Huon, 2005; Fehm and Schmidt, 2006); MPA manifests through three distinct avenues: cognition, autonomic arousal, and behavior (Kenny, 2005).

The hypothesis of optimal performance involves a moderate level of arousal (Yerkes and Dodson, 1908), however, persistent fear and high levels of anxiety can damage performers’ mental health and overall wellbeing (Fishbein et al., 1988; Kenny et al., 2012). Moreover, musicians can suffer from mental illness (Brodsky, 1996), which adds another level of psychological vulnerability. According to a survey of 2212 professional musicians from American orchestras, 24% reported MPA, 13% reported acute anxiety, and 17% suffered from depression (Fishbein et al., 1988). In addition, females and young musicians (<30 years) are particularly vulnerable to MPA and other mental illness based on a survey of Australian musicians (Kenny et al., 2012).

Music psychologists have devoted to exploring coping strategies to reduce MPA in higher education (Clark and Williamon, 2011; Osborne et al., 2014). According to two systematic reviews of interventions aimed at reducing MPA, a combination of two or more types of therapy produced the most effective result (Brugués, 2011; Goren, 2014). However, research involving MPA interventions has often been conducted using methods that require multiple psychological sessions or special technology and materials to be successful. Therefore, it is important to explore methods that are simple and that potentially produce immediate effects in terms of decreasing the impact of MPA in real-world music-performance contexts.

Expressive writing has been used as a therapeutic intervention for reducing personal stress and anxiety, and for encouraging healing from a traumatic experience for decades (Pennebaker and Beall, 1986; Pennebaker et al., 1988; Baikie and Wilhelm, 2005; Pennebaker and Smyth, 2016). Extensive research has indicated that expressive writing produces substantial immediate and long-term benefits in physical and mental health (King, 2002; Beckwith et al., 2005; Frattaroli, 2006). To date, although many of these studies have been conducted with college student populations, the research has focused on alleviating test anxiety across non-music disciplines (Frattaroli et al., 2011; Ramirez and Beilock, 2011; Park et al., 2014).

Similar to MPA, test anxiety refers to a negative response to an exam situation which manifests both physically (e.g., increased heart rate, nausea) and cognitively (e.g., self-doubt, feeling unprepared or not expert enough; Cassady and Johnson, 2012). Research has found that anxious thoughts take up valuable working memory and interfere with concentration (Moran, 2016). In line with this finding, music psychology research have found that during a music performance, students who performed poorly reported more disturbing thoughts and worries, especially immediately before they froze in the middle of their performances (Oudejans et al., 2017).

To alleviate test anxiety, Ramirez and Beilock (2011) developed an intervention based on expressive writing that is particularly effective in decreasing the impact of test anxiety on a math test. Students wrote about their thoughts and feelings regarding the upcoming math exam immediately before the exam took place. Their results showed that students with high-math test anxiety improved their grades from B− to B+. Another study reported a similarly positive effect of an expressive writing intervention on applicants taking medical and law school entrance exams (Frattaroli et al., 2011). Participants had significantly higher test scores and significantly lower pre-exam depressive symptoms than the control group. Although the mechanism underlying the EWI’s therapeutic effect is unclear (Danoff-Burg et al., 2010), the hypothesis is that EWI helps to regulate the fear and anxiety by giving students an opportunity to express their anxious thoughts, and re-evaluate the situation before an impending test (Ramirez and Beilock, 2011).

The aim of the current study was to investigate whether an expressive writing intervention (EWI) effectively reduces MPA and measurably improves performance outcomes in college-level piano students. We also explored the factors that determine who would benefit from EWI the most.

## Materials and Methods

### Participants

College students majoring in music (*N* = 35) participated in this study. Participants were either piano performance majors (*n* = 16) or group/secondary piano students (*n* = 19)^1^. The age of participants ranged from 18 to 61, (*M* = 20.90, *SD* = 4.32). For piano major students, the average years of playing piano were 14.6 years and the average practice time was 23.1 h per week. Piano students’ average lesson time was 1 h (individual session) a week. For group/secondary piano students, the average years of playing piano and their primary instrument were 2.47 and 8.91 years, respectively. The average piano practice time (individual session) was 3.2 h a week. Secondary piano students’ average piano lesson time was 2–3 h per week (group class).

### Study Design

To induce the proper MPA, two performing tasks were designed for each group according to their piano curriculum and exam scope. Sixteen advanced piano students were asked to perform a solo piano work of their own choice from memory and nineteen secondary piano students were asked to sight-read a piano music selection without prior practice.

Piano major students performed the same piano piece in both performances (baseline and post-EWI) and the pieces ranged from 4 to 18 min long. Baseline performances took place in students’ weekly studio performance class where they regularly play in front of peers and are critiqued by a piano professor. The post-EWI performance took place under various performance settings, including a studio performance class, a piano area noon recital, and a piano jury.

For group/secondary piano students, their piano instructors chose two different pieces at the appropriate reading level for them to sight-read. During the baseline sight-reading performance, each student was given an eight-measure piano musical selection to play by sight. The baseline sight-reading performance was conducted in a regular classroom and students were not graded on this performance. During the post-EWI performances, students played a new sight-reading selection at their mid-term piano tests; therefore, the students knew that they would receive a grade on the performance. The author and the secondary piano class instructor were presented in the room.

### Procedure

**FIGURE 1:**
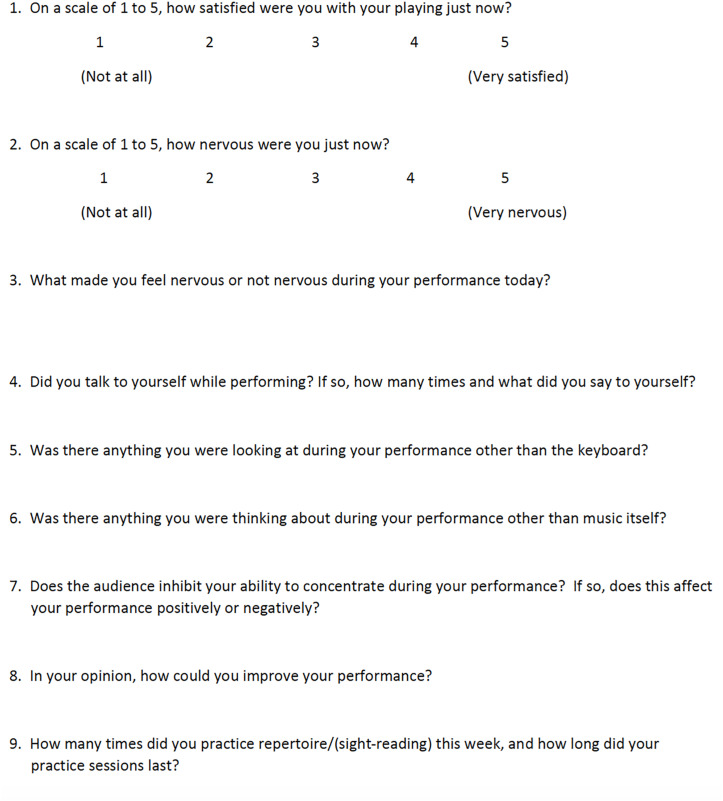
Post-repertoire/sight reading performance self-report.

Prior to the experiment, a questionnaire of students’ music educational experience and performance habits that adapted from Roland (1993) was administered. The experiment consisted of two phases: a baseline and post-EWI performance, occurring 2 to 3 days apart. Students were given a piece of paper to complete the writing exercises during the post-EWI performance. We videotaped each student’s two performances (baseline and the post-EWI) and obtained pulse rates using a pulse oximeter immediately before each performance. At the end of each performance, participants were immediately asked to fill out a post-performance self-report questionnaire (Figure 1).

### Writing Exercises

During the post-EWI performance, students were randomly assigned to one of two groups: the expressive writing group and the control group. The expressive writing group wrote about their current feelings and thoughts regarding the upcoming performance; the control group wrote about an event that happened recently. They were given 10 min to write. Their performances began right after they finished the writing exercises.

### Measures

#### Performance Quality

Students’ performance recordings were analyzed acoustically using a form adapted from Gruson’s (1988) Observational Scale for Piano Practicing (OSPP). The OSPP is used to analyze playing errors such as repetition of notes or sections, pauses, and/or wrong notes (Table 1). Table 2 contains the explanation of each term used for analysis. Three independent judges graded each performance recording according to OSPP. The judges were not told that they were grading the recordings of the same subjects or the performance order (whether pre- or post-EWI). We took the average grades from three judges to produce a single performance quality score for each student.

**TABLE 1:** Analysis form for each recording.

	1st recording	2nd recording
Re-start		
Re-note/section		
Wrong note		
Omission		
Hesitation		
Total errors		
Pause		

**TABLE 2:** Explanation of each term.

Pause	Stop playing for at least 1 s and create a gap between the notes
Re-start	Repeat the whole piece from the beginning
Re-note/section	Repeat a note/section on which an error may/may not have occurred with or without correction
Wrong note	Play incorrect notes
Omission	Omit note or section of the piece
Hesitation	Slow the tempo beyond the range permitted by the music

#### Pre-performance Pulse-Rate

Each student’s pulse rate was recorded immediately before baseline and post- EWI performances. Two of their pre-performance pulse rates were compared to measure levels of arousal such as anxiety.

#### Questionnaire

Only three questions that were relevant to the current study were included in the results analysis:

1. Have you received any training or information on how to deal with performance anxiety during your studies?
2. In which performance settings are you most likely to feel very anxious?
3. In general, do you talk to yourself right before and during your performance? If yes, please specify the content of your talk.

#### Anxiety and Satisfaction

Performance anxiety and satisfaction were reported by each participant immediately following their performance. Each item was rated on a 5-point scale ranging from 1 (“not anxious/satisfied at all”) to 5 (“very anxious/satisfied”).

#### Post-performance Questions

Three questions were used to evaluate the influence of the EWI on student anxiety level and performance quality.

1. What made you nervous or not nervous for today’s performance?
2. Did you talk to yourself as you were performing, if so, how many times and what did you say to yourself?
3. In your opinion, how could you improve your performance?

## Results

### Pre-intervention Analysis

A series of independent samples *t*-tests were conducted to compare the group differences across the expressive writing and control group at baseline. There were no statistically significant differences between the two groups of advanced piano players in age, *t*(14) = 0.93, *p* < 0.36, years of piano study, *t*(14) = 0.76, *p* < 0.46, or weekly practice times, *t*(14) = 1.35, *p* < 0.19. For secondary piano students, similarly, there were no statistically significant differences between the two groups in age, *t*(17) = 1.23, *p* < 0.23, years of piano study *t*(17) = 0.05, *p* < 0.96, or weekly piano practice times, *t*(17) = 1.76, *p* < 0.09.

For this study, all 35 participants completed both baseline and post-EWI performances, however, we were not able to record three participants’ pulse rates during the baseline test; therefore, only 32 out of 35 participants’ pulse rates were included in the analysis of results.

### Overall Intervention Efficacy

#### Performance Errors of Both Performance Tasks

**FIGURE 2:**
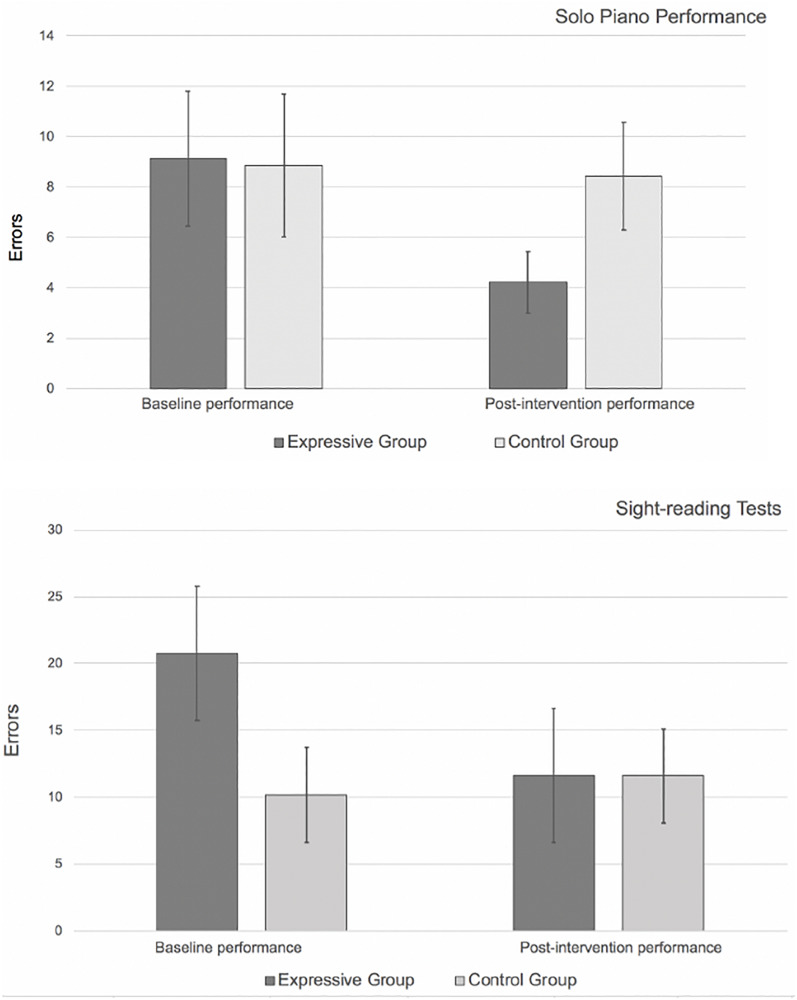
Mean performance errors by both performance types and conditions (bars show standard error).

The overall performance errors were analyzed using a mixed-design ANOVA to compare the within-subject factor time (pre, post), and two between-subject factors: the intervention group (expressive writing, control), and performance type (solo piano, sight-reading). A significant interaction between time and the intervention group, *F*(1,31) = 9.91, *p* < 0.004, indicated that the expressive writing group demonstrated a significant decrease in errors (mean decrease = 7.3), whereas the control group performances remained the same before and after the intervention (Figure 2).

#### Expertise Level and Effectiveness of EWI

Importantly, while the level of piano-playing experience was different between the two groups (piano major students = 14.6 years, secondary piano students = 2.47 years), the impact of the intervention on errors was similar for both groups, *F*(1,31) = 1.63, ns, suggesting that the impact of the intervention on errors did not depend on the level of experience. For both expressive writing groups (solo and sight-reading), performance errors decreased by approximately 50% in the post-EWI performance when compared to baseline performances (Table 3).

**TABLE 3:** Mean performance errors by both type and condition (standard deviation in parentheses).

	Piano solo performance (*N* = 16)		Sight-reading test (*N* = 19)	
	Expressive group (*n* = 9)	Control group (*n* = 7)	Expressive group (*n* = 14)	Control group (*n* = 5)
Baseline	9.11 (8.02)	8.85 (7.49)	20.79 (12.4)	10.2 (7.95)
Intervention	4.22 (3.63)	8.42 (5.68)	11.64 (7.4)	11.6 (7.83)

#### Other Measurements

In contrast to performance errors, no significant statistical differences in pulse rate, self-reported satisfaction, or self-reported performance anxiety were observed before and after the intervention across performance types.

### Comparing High Versus Low Self-Talk Groups

In order to identify students who are most likely to benefit from EWI, we further analyzed our data. Within the experimental group, we found large differences in the frequency of organic self-talk during performance. In addition, the content of these self-conversations was different. According to students’ self-reports, high self-talkers (HSTs) were more involved in emotional conversations (examples: “I had this inner monolog running to express my anger at my mistakes when they really happened,” “how could I have made the same mistake in the same place again?”). In contrast, low self-talkers (LSTs) reported that they were either not aware of any self-talk, or only had a few internal instructional conversations during the performance (examples: “before chord changes, I talked to myself about where I needed to move my hands,” “I am taking a breath and not being fluid”). Based on the theoretical assertion that “self-talk lies at the core of anxiety” (Conroy and Metzler, 2004, p. 69), and the assumption that excessively frequent self-talk may interrupt the performer’s concentration and disrupt execution (Zinsser et al., 1998), we hypothesized that the HST group would gain the most benefits from the expressive writing intervention due to high levels of performance anxiety.

Two subgroups collapsed across performance type were created: participants (*n* = 8) reported frequent self-talk during performance, and participants (*n* = 8) who reported little or no self-talk during performance. These sixteen participants’ data were studied further^2^.

The baseline performance data between the HSTs and LSTs were compared by the *t*-test in the four following measures: performance errors, pulse rate, self-reported anxiety levels, and performance satisfaction. A preliminary *t*-test score indicated significant differences in self-reported performance anxiety levels between the HSTs and the LSTs, *t*(16) = (2.31), *p* < 0.03. This confirmed that the HSTs felt more anxious in the baseline performance, however, the other three measures (performance errors, pulse rate, and performance satisfaction) did not demonstrate significant statistical differences. It is worth noting that in the baseline performance, the HSTs demonstrated approximately 10% higher pulse rates, 46% more performance errors, and reported 26% less self-satisfaction on average when compared with the LSTs in raw data (Table 4).

**TABLE 4:** Mean comparison scores by group and condition (standard deviation in parentheses).

	Low self-talk group (*n* = 8)		High self-talk group (*n* = 8)	
	Baseline	Intervention	Baseline	Intervention
Pulse Rate*	94.00 (8.58)	95.8 (9.29)	104.87 (14.9)	95 (15)
Errors	11.13 (8.23)	7.38 (7.89)	20.63 (14.91)	8.13 (6.17)
PA level	2.56 (0.82)	2.50 (1.30)	3.68 (1.09)	2.50 (1.19)
Satisfaction	3.00 (1.06)	3.75 (0.70)	2.37 (1.27)	3.56 (0.82)

*Pulse rate excluded one participant in the HST group (n = 7), due to no record for the baseline performance.

There was a clear gap between the HSTs and LSTs in the baseline performance. To examine the impact of EWI on these two groups, we compared baseline and post-EWI performance data in the following measures: performance errors, pulse rates, self-reported anxiety levels, and performance satisfaction. Measures were analyzed separately using a 2 × 2 mixed-design ANOVA to compare the within-subject factor time (pre, post), and the between-subjects factor self-talk (HSTs vs. LSTs) within the expressive writing group.

#### Performance Errors

**FIGURE 3:**
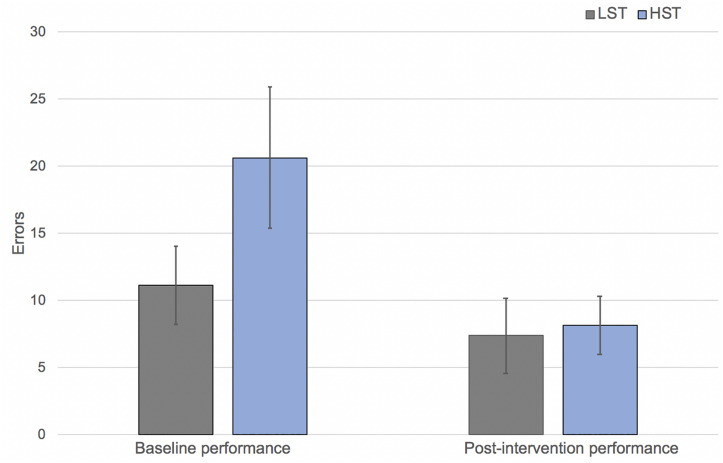
Mean performance errors by both group and condition (bars show standard error).

A significant interaction was seen between performance time (pre, post) and group (HST or LST), *F*(1,14) = 5.82, *p* < 0.03, indicating that the HST group demonstrated a significant decrease in performance errors (mean decrease = 12.5, *SD* = 8.74), whereas the LST group did not demonstrate a statistically significant difference in performance errors before and after the EWI (mean decrease = 3.75, *SD* = 7.02) (Figure 3).

#### Other Measures

For pulse rate, there was a significant interaction between performance time and group (HST or LST), *F*(1,14) = 14.65, *p* < 0.002, indicating the HST group had a significant decrease in pulse rate (mean decrease = 9.88), whereas pulse rate of the LST group remained the same. In self-reported performance anxiety levels, there was a significant interaction between performance time and group, *F*(1,14) = 4.99, *p* < 0.04, indicating the HST group demonstrated a significant decrease in the pre-performance anxiety levels (mean decrease = 1.18), whereas the LST group did not show change (mean decrease = 0.06). In contrast, no significant interaction was found in self-satisfaction, although there was a main effect for self-satisfaction, *F*(1,14) = 15.6, *p* < 0.001. This indicated both groups showed increased satisfaction in the post-EWI performance.

#### Self-Talk

The frequency of self-talk was significantly reduced in the HST group. Only three out of eight participants reported that they still engaged in self-talk during the post-EWI performance. Among them, two participants reported that they self-talked only a few times when they made a mistake. The LST group results remained unchanged; only one participant reported that he coached himself through the music.

## Discussion

### Support for EWI as an Effective Intervention for Music Performance Anxiety

This is the first empirical study that we know of to use expressive writing intervention to reduce music performance anxiety and improve performance in college-level piano students. The data from our study supports the positive results in the reduction of performance anxiety with expressive writing intervention for college-level piano students resulting in improved performance outcomes.

In our study, college-level piano students (both solo players and sight readers) who received an expressive writing intervention demonstrated significant overall performance improvement. In post-expressive writing intervention performances, marked reduction in performance errors occurred in the experimental condition for 15 of the 23 participants. Results showed a 50% reduction in performance for both performing tasks (performing a solo piece and performing a sight-reading test) compared to the baseline performance. The control group experienced no major changes between the two performances.

We also found that the writing length and details may affect the impact of an expressive writing intervention. A previous study suggested that emotional expression and narrative structure are the two key factors that determine the efficacy of expressive writing intervention (Danoff-Burg et al., 2010). In the present study, two students in the expressive writing group wrote only two sentences in the writing exercise. No major changes were found in their performance outcomes and other measurements between the two performances.

Previous studies suggest that performance outcomes may improve organically through the accumulation of practice sessions (Ericsson et al., 1993; Ericsson and Anders, 2006), however, we found that within a short period of 1 to 3 days, students in the control group (comprised of both solo piano players and sight-readers) did not improve performance quality significantly with regular practice. Instead of making fewer errors, 6 of the 12 students in the control group performed slightly less well during their second performance; 2 students’ performance outcomes remained the same, and 4 performed better. The implication of these results is that practicing the night before a recital or test may not significantly improve performance quality the following day. In piano performance, enhancing technique and improving performance quality is a relatively long process.

While differences lie in the level of piano-playing experience between the piano major and group piano students, both groups benefited from writing their thoughts and feelings prior to a performance. This suggests that the level of experience of the student does not influence the efficacy of expressive writing intervention. Furthermore, we identified performance anxiety as a critical variable that predicted the positive impact of expressive writing intervention. Compared with less anxious peers, the students (including both solo performers and sight-readers) who experienced the highest anxiety at baseline performance benefited the most from the expressive writing, as evidenced by more significant reductions in performance errors, self-reported anxiety levels, and pulse rates in post-expressive writing intervention performance.

### Exploring the Relationship Between Self-Talk and Performance Anxiety

Another finding of our study was a close association between performance anxiety and frequency of self-talk during the baseline performance. Piano students who engaged in frequent self-talk during the baseline performance reported 30% higher performance anxiety levels than students who reported little or no self-talk. This finding is consistent with Conroy and Metzler’s (2004) cognitive theories on anxiety, suggesting that “self-talk lies at the core of anxiety” (p. 69). Organic self-talk is often portrayed as “the primary pathology or dysfunction” of anxiety (Beck et al., 1985, p. 85). The intrusive thoughts included in organic self-talk cause distraction, which may threaten performance outcomes. Furthermore, we found that frequent self-talkers had faster pulse rates, consistent with Rushall et al. (1988) study of cross-country ski performance. The authors reported that pulse rates were higher in self-talk conditions than in the control condition that did not include self-talk. Tod et al. (2011) suggest that the impact of self-talk on performance may be mediated by physiological changes in arousal, as reflected in variations in pulse rates.

Our study shows the impact of negative self-talk specifically associated with music performance anxiety is consistent with previous research (McKellar et al., 1996; Treadwell and Kendall, 1996; Ronan and Kendall, 1997; Hiebert et al., 1998; Muris et al., 1998), which has demonstrated that negative self-talk is positively associated with anxiety between normal and clinically anxious children, as well as with college students. Academic performance anxiety has also been strongly associated with negative self-talk during exams (Hunsley, 1987; Fernandez and Allen, 1989). Similarly, in our study, piano students who were frequent self-talkers reported that they were occupied by negative self-talk, such as expressing anger or guilt over performance mistakes. Expressive writing intervention led to a measurable reduction in self-talk for 5 of 8 frequent self-talkers, who reported no incidence of self-talk during the post-expressive writing intervention performance. Among three participants who reported ongoing self-talk during expressive writing intervention performances, there were changes in the focus of their self-talk from emotional, harsh self-criticism to instructional self-talk that presented strategies for getting back “on track.” In addition, these frequent self-talkers reported lower levels of anxiety during post-intervention performances. Our study supports Kendall and Treadwell’s (2007) findings that reducing anxious self-talk results in less anxious states and positively influences performance outcomes.

### Self-Talk’s Content

Self-talk is often considered an effective strategy for improving athletic performance (Tod et al., 2011; Webb et al., 2012), however, the content of self-talk must be planned and manipulated systematically in advance. Besides differentiating self-talk as positive versus negative, previous research has categorized self-talk in athletes as motivational (e.g., “give it all”) versus instructional (e.g., “push the ball”). These types of self-talk may have different effects on specific task performances (Theodorakis et al., 2000; Hatzigeorgiadis et al., 2004). Instructional self-talk may be more effective for tasks requiring fine motor skills, such as dexterity, hand-eye coordination, precision, and accuracy in sports (e.g., dart throwing, golf-putting, and shooting a basketball), and by extension, perhaps piano performance. In contrast, motivational self-talk may be more effective for tasks requiring strength and endurance like weightlifting and long-distance running.

In this study, we did not manipulate the content or the type of students’ self-talk. Nevertheless, when we compared the content of self-talk between baseline and post- expressive writing intervention performances, we found significant changes in content and focus. After taking the expressive writing intervention, self-talk became less subjective and more instructional oriented. For example, in baseline performances, students stated, “shock at the level of my anxiety” or “I was telling myself how uneven it was.” These students either engaged in negative self-evaluation or focused on negative emotional thoughts while performing. After expressive writing intervention, examples of their reported self-talk content included comments about the instrument: “this piano has a firm touch” or “(to) get back somewhere near where I fell off.” These talks became less judgmental and more objective. We hypothesize that writing feelings and thoughts before a performance can regulate emotions, and redirect attention to music-related or more objective thoughts. Instead of training people to remember and use pre-planned self-talk cues and strategies during a performance, expressive writing intervention may produce similar results in more organic and potentially effective ways. This possibility will require further investigation.

We also noticed that all piano students reported that they usually talk to themselves before a performance in positive ways such as “everything will be fine” or even reciting a psalm from the Bible. Perhaps these habits demonstrate that everyone experiences emotional arousal prior to performing. During a performance, however, only the students with the high levels of music performance anxiety reported using frequent self-talk, engaging mostly in the negative commentary: “That was bad!” or “how could I made the same mistake in the same place again?” However, the less anxious students reported little or no self-talk. In our study, expressive writing intervention appears to provide piano students with a mechanism for staying focused and eliminating unnecessary negative or self-critical conversations while performing.

### Future Research on Music Performance Anxiety

Our findings regarding the connection between various forms of self-talk and performance anxiety, and the impact of expressive writing intervention on the amount and quality of self-talk, is intriguing and promising. However, it requires further study.

Neuroimaging studies have found that math anxiety is associated with hyperactivity in right amygdala regions that are responsible for processing negative emotions, and reduced activity in posterior parietal and dorsolateral prefrontal cortex regions are responsible for working memory processes (Young et al., 2012). We expect that piano students may show similar patterns of brain activation when experiencing music performance anxiety. In addition, we found that most of our participants reported moments when their minds went blank, or they had memory slips that lasted only a few seconds while they were performing. Since the amygdala is also linked to the hippocampus, which is known to be critical for long-term memory (Bird and Burgess, 2008; Gazzaniga et al., 2009, 378–423), one may hypothesize that hippocampal activity may be decreased when a piano student’s mind “goes blank” on stage. In order to provide further insight into this process, future research might include functional magnetic resonance imaging (MRI) to identify which parts of the brain are associated with memory slips during music performance or the changes in patterns of activation associated with post-expressive writing intervention performance.

### Limitations

The length of solo piano repertoires was varied, though there is no correlation between the pieces’ lengths and the students’ performance quality. Future studies may consider the length of the solo piano repertoires as a factor when recruiting the participants. Also, the number of participants in the sight-reading’s control group was limited. A larger sample would have provided greater statistical evidence. Additionally, the performance settings in our study did not necessarily induce stress for all participants. The most stressful performance settings in this study, as ranked by our participants (*N* = 35) were solo/small ensemble recitals (81%) and public masterclasses (75%). The fear of being criticized by prestigious musicians, teachers, or a public audience may increase performance anxiety. Since the expressive writing intervention appeared to be most effective for students with high performance anxiety, it would be meaningful to test this expressive writing intervention in more high-stakes performance settings. We also did not officially ask our participants to give feedback about the expressive writing intervention. Participants’ feedback will be valuable; although the participants may not be able to accurately evaluate the effects of the expressive writing intervention or may not be aware of what caused performance quality changes.

## Conclusion

Unlike professional athletes, musicians are often left alone without resources or strategies to help them to cope with performance anxiety. Most piano students are unaware of either their need for psychological support or strategies to help combat performance anxiety. Our findings address an urgent need in current music pedagogy to address performance anxiety. Seventeen of 35 students in our study stated they had never received any education about how to identify and cope with performance anxiety. When we asked students how they could improve their performances, most of them answered that they needed to spend more time practicing. Only a few participants indicated that they might need to practice coping with their performance anxiety. Our study suggests a better understanding of performance anxiety, and the use of expressive writing intervention may help establish appropriate pedagogical support for music students.

In conclusion, this study largely confirms our hypothesis that an expressive writing intervention could effectively help piano students cope with performance anxiety. Students who were highly anxious about performing received the most benefit from this intervention. This small but innovative tool was helpful in reducing the performance gap between the most anxious students and least anxious students and can be seen as a potentially useful procedure for all music performers.

## Data Availability Statement

All datasets generated for this study are included in the article/supplementary material.

## Ethics Statement

The studies involving human participants were reviewed and approved by the University of Arizona, Fred Fox School of Music. The patients/participants provided their written informed consent to participate in this study.

## Author Contributions

YT and LR contributed the design of the study and performed the statistical analysis. YT organized the database and wrote the first draft of the manuscript. Both authors contributed to manuscript revision, read, and approved the submitted version.

## Conflict of Interest

The authors declare that the research was conducted in the absence of any commercial or financial relationships that could be construed as a potential conflict of interest.

## Footnotes

1 Secondary piano class is required curriculum at the university level for non-keyboard music students throughout the United States. Students usually go to the piano class as a small group in a keyboard lab. They are expected to develop keyboard proficiency and musicianship through the study of scales and arpeggios, chord progression, solo repertoire, and other fundamentals.

2 Please note, only 15 participants’ pulse rates were compared, because one participant’s pulse rate data in the LSTs was missing.
